# The Medical Union

**Published:** 1891-01

**Authors:** 


THE MEDICAL SOCIETY OF THE STATE OF NEW YORK.—The officers of this Society are actively at work preparing for the next annual meeting, which will be held in Albany on Tuesday, Wednesday, and Thursday, February 3, 4, and 5, 1891. The indications are that this will be a most interesting and profitable meeting from a scientific standpoint, as a number of papers of more than ordinary interest have already been offered. The conspicuous prominence that the Society holds with reference to reform in medical education, and the important action to be taken with regard to the State Board of Medical Examiners, will, undoubtedly, insure a large attendance. The county societies should look to it that they are represented with a full quota of delegates.

---

THE MEDICAL UNION held its regular monthly meeting at The Genesee on Tuesday evening, December 16, 1890. The paper of the evening was presented by E. C. Waldruff. Subject: Electrolysis in Urethral Strictures. After the regular business of the evening, the annual election of officers took place, resulting as follows: President, Samuel G. Dorr; vice-president, W. C. Phelps; secretary-treasurer, DeWitt C. Greene.

